# Reviewed article: Rosa RS, Menezes JS, Martinez EZ, Boery RNSO, Ribeiro IJS, Contreras JCZ, Junior JB, Mussi RFF, Moreira SLF, Lemos GS, Santos ISC. Factors associated with inadequate blood pressure control among hypertensive individuals living in a Quilombola Community: a cross-setional study, Brazil, 2017-2018. Epidemiol Serv Saude. 2025:34;e20240556

**DOI:** 10.1590/S2237-96222025v34e20240556.b

**Published:** 2025-10-03

**Authors:** Cristine Vieira do Bonfim

**Affiliations:** 1 Fundação Joaquim Nabuco, Recife, PE, Brazil Fundação Joaquim Nabuco Recife PE Brazil

## First round comments

Estimados(as) autores e autoras,

O manuscrito intitulado “Fatores associados ao controle pressórico em hipertensos residentes em uma comunidade quilombola (RESS-2024-0556) apresenta um tema relevante para a saúde pública, em especial, por estudar a população quilombola. Trata-se de um estudo transversal que teve por objetivo identificar a prevalência e fatores associados ao controle pressórico em hipertensos residentes em uma comunidade quilombola. Os(as) revisores(as) apontaram preocupações relevantes, sobre a coleta dos dados, a imprecisão na definição do desfecho analisado e das variáveis mensuradas. A forma de análise de dados precisa ser descrita mais detalhadamente e os resultados evidenciar quais são os fatores associados. Acrescente-se que a discussão necessita de aprofundamento e retirada dos dados numéricos de resultados. Seria muito importante evidenciar a contribuição do estudo para a população quilombola. Comentários mais detalhados são realizados abaixo.

Comentários maiores

O título necessita refletir com exatidão o conteúdo do estudo. Nesse sentido, sugere-se a inclusão do local de realização do estudo, o período de referência e o desenho do estudo.

No resumo, nota-se a ausência de informações necessária para uma melhor compreensão do estudo. Sugere-se informar somente o delineamento do estudo. Incluir a forma de coleta de dados, as variáveis estudadas e os métodos de análise de dados aplicados para a identificação dos fatores associados. Na seção de resultados, são apresentadas variáveis que devem ser deslocadas para a seção de métodos. Seria interessante colocar o número total de observações nos resultados, retirando da seção de métodos. Um ponto importante nos resultados é evidenciar quais foram os fatores associados. As conclusões precisam responder diretamente ao objetivo do estudo.

A introdução necessita de um aprofundamento teórico, em relação aos fatores que serão analisados.

Sugere-se a inclusão da justificativa para a realização do estudo. A afirmativa de que “são poucas as evidências voltadas ao controle pressórico em pessoas destas populações”, necessita da inclusão de uma referência.

Na seção de métodos, a população do estudo é o número real de registros e não uma estimativa.

Sugere-se retirar o nome do projeto ao qual o estudo se vincula, uma vez que essa informação irá ser apresentada no item de considerações éticas.

A informação sobre as entrevistas está deslocada no item de desenho e população. Sugere-se que seja apresentada no item coleta de dados.

No item “variáveis” é importante apresentar as variáveis e as suas categorizações.

A tabela 1 apresenta a análise do controle de pressão, classificado como inadequado. Essa classificação precisa ser explicitada de forma bem evidente e compreensível na seção de métodos.

A coleta de dados precisa ser descrita com mais detalhes.

Na análise de dados, a distribuição de frequências e a estatística descritiva com as medidas de tendência central e dispersão aplicadas, precisam ser descritas.

Boa parte da análise de dados, descrita nesse item, não foi localizada na seção de resultados.

No item de resultados, é relevante incluir as frequências absolutas, das quais as relativas foram derivadas.

A seção de resultados, necessita ser revisada, para que sejam retiradas as interpretações.

Um aspecto importante na seção de resultados é evidenciar quais são os fatores associados identificados no estudo.

Na seção de discussão tem a seguinte informação “pessoas com alta adesão ao uso de medicamentos”. Essa variável não foi descrita na seção de métodos, para que seja a compreensão da sua estratificação.

A seção de discussão necessita ser revisada, para que sejam retirados os dados numéricos dos resultados. Importante revisar a seção com atenção, pois em alguns momentos tem-se a sensação de que os resultados estão sendo novamente descritos, de uma outra forma.

A seção de referências necessita de atualização.

Comentários menores

O quarto parágrafo da introdução é formado por uma única frase. Sugere-se que a redação seja realizada com frases mais reduzidas, para facilitar a melhor compreensão da argumentação.

Sugere-se que sejam evitadas citações múltiplas na introdução e somente as principais referências sejam citadas.

Página 4, linha 12, essa é uma afirmativa bastante importante para o artigo. Dessa forma, sugere-se a inclusão de uma referência atualizada.

Recommendation: Reject

## Second round comments

Estimadas autoras e estimados autores,

Agradecemos o envio da versão revisada do manuscrito intitulado “Fatores associados ao controle pressórico em hipertensos residentes em uma comunidade quilombola” (RESS-2024-0556.R1). Solicitamos que seja encaminhada a carta com a descrição ponto-por-ponto das modificações realizadas no manuscrito na primeira rodada de revisão. Todas as adequações propostas devem ser consideradas e as alterações devem ser realçadas no documento, tornando-se necessária a resposta a todos os comentários e sugestões dos(as) revisores(as). As sugestões que não forem consideradas deverão ser justificadas. Sugere-se utilizar a ferramenta de marcação de alterações. Para que os(as) autores(as) possam enviar a versão revisada com as marcações de alterações e a carta para os editores com a resposta ponto-por-ponto, estamos solicitando uma revisão menor.

Recommendation: Minor revision

## Third round comments

Estimados autoras e autores,

Agradecemos o envio da carta com as respostas aos comentários item por item. Solicitamos, gentilmente, que as sugestões atendidas, sejam realçadas. A ferramenta “Controlar alterações” (na aba “Revisão” do Word) deverá ser aplicada para alterações realizadas no manuscrito. As sugestões que não forem consideradas deverão ser justificadas com fundamentação científica. A versão reformulada do manuscrito deve considerar as instruções aos autores da RESS e os itens de padronização indicados na revisão técnica. Para realização desse ajuste, estamos solicitando uma pequena revisão. Agradecemos, novamente, o seu interesse pela RESS. 

Recommendation: Minor revision

## Fourth round comments

Estimadas autoras e estimados autores

Agradecemos o envio do manuscrito intitulado “Fatores associados ao controle pressórico em hipertensos residentes em uma comunidade quilombola” e o empenho na realização da revisão.

No entanto, todos os comentários e sugestões dos(as) revisores(as) e do Núcleo Editorial devem ser considerados e respondidos, item por item, em carta aos editores. Devem especificadas as páginas, parágrafos e linhas onde as modificações foram realizadas. Para as sugestões que forem atendidas, deverá ser realçada a alteração realizada no manuscrito utilizando-se o amarelo. As sugestões que não forem consideradas deverão ser justificadas com fundamentação científica.

A versão reformulada será novamente avaliada pelo Núcleo Editorial, considerando-se as instruções aos autores da RESS.

Por favor, não deixem para ressubmeter no último dia, pois o sistema não é nacional e sempre irá expirar o prazo no dia limite.

Aguardamos o envio do manuscrito com as alterações destacadas e da carta com a descrição das alterações realizadas.

Recommendation: Minor revision

## Fifth round comments

Estimados autores e estimadas autoras,

Agradecemos o envio do manuscrito revisado. O Núcleo Editorial da revista Epidemiologia e Serviços de Saúde (RESS) considera o tema do manuscrito intitulado “Fatores associados ao controle pressórico em hipertensos residentes em uma comunidade quilombola” relevante e tem interesse em publicar artigos a ele relacionados.

Estamos na quarta rodada de revisão, e solicitamos nas anteriores que enviem o manuscrito com as alterações destacada em amarelo. Igualmente, solicitamos, o envio da carta respondendo item-por-item aos pareceres. Observamos que os destaques não foram realizados no manuscrito. Dessa forma, informamos que essa será a última rodada de avaliação.

Todos os comentários e sugestões do Núcleo Editorial devem ser considerados e respondidos, item por item, em carta aos editores. Devem especificadas as páginas, parágrafos e linhas onde as modificações foram realizadas. Para as sugestões que forem atendidas, deverá ser realçada a alteração realizada no manuscrito utilizando-se o amarelo. As sugestões que não forem consideradas deverão ser justificadas com fundamentação científica. Nesse sentido, estamos solicitando uma pequena revisão para realização desses ajustes.

A versão reformulada será novamente avaliada pelo Núcleo Editorial, considerando-se as instruções aos autores da RESS.

Solicitamos, gentilmente, que não deixem para ressubmeter no último dia, pois o sistema não é nacional e sempre irá expirar o prazo no dia limite.

Aguardamos o envio do manuscrito com as alterações destacadas e da carta com a descrição das alterações realizadas.

Recommendation: Minor revision

## Sixth round comments

Estimadas autoras e estimados autores

O Núcleo Editorial da RESS informa que, após a avaliação, o manuscrito intitulado “Fatores associados ao controle pressórico inadequado de hipertensos residentes em comunidade quilombola no Nordeste do Brasil, de 2017 a 2018: estudo transversal” (RESS-2024-0556R5) foi considerado com potencial para publicação, mas reformulações maiores são necessárias. Todos os comentários e sugestões do Núcleo Editorial devem ser considerados e respondidos, item por item, em carta aos editores(as). Para as sugestões que forem atendidas, deverá ser realçada a alteração realizada no manuscrito, utilizando-se o amarelo. As sugestões que não forem consideradas deverão ser justificadas com fundamentação científica. A versão reformulada do manuscrito deve considerar as instruções aos autores da RESS e os itens de padronização indicados na revisão técnica. Solicita-se, gentilmente, que os(as) autores(as) não deixem para enviar o manuscrito no último dia, pois o sistema não é nacional e sempre irá expirar o prazo no dia limite.

Aguardamos o envio do manuscrito com as alterações destacadas e da carta com a descrição das alterações realizadas.

Cordialmente,

Núcleo Editorial

Título - ok

Resumo

Resumo não deve conter siglas/abreviaturas.

O número de observações deve ser deslocado da seção de métodos para os resultados.

A seção de métodos descreve técnicas estatísticas que não observamos os resultados. Deve ocorrer uma correspondência entre métodos e resultados.

Os resultados devem evidenciar claramente quais são os fatores associados encontrados. Solicitado novamente.

A conclusão deve responder diretamente ao objetivo. Vulnerabilidade não foi uma categoria analisada, não se encontra nos resultados, logo não é possível contemplá-la na conclusão.

Descritores: incluir o desenho do estudo.

Introdução

De forma geral, a introdução necessita ser revisada para incluir dados epidemiológicos sobre o tema da hipertensão. Revisar para que o texto informado no artigo seja fidedigno à informação de cada uma das referências citadas. Para os estudos citados sempre informar o local do estudo e o período. Observa-se a presença de várias frases sem as referências que as fundamentam. A seção está extensa e apresenta informações repetidas. Precisa ser revisada para que seja mais concisa.

Página 4, primeiro parágrafo. A afirmativa feita na segunda, frase não foi localizada no artigo citado.

Página 4, segundo parágrafo. Substituir “altamente prevalente”, por dados que demonstrem essa prevalência. A afirmativa feita necessita de citações. Última frase desse parágrafo necessita de referências. Acrescente-se que parece ser uma informação repetida, semelhante à da primeira frase.

Página 4, terceiro parágrafo. Quais são os desafios? Especificar e citar as referências que fundamentam tal afirmativa. As frequências relativas somente fazem sentido, quando acompanhadas das absolutas das quais foram derivadas. Incluir a frequência absoluta.

Página 4, linha 29, são as pessoas negras ou as pessoas com reduzido poder aquisitivo? Essa afirmativa também necessita ser acompanhada pelas referências que as fundamentam.

As referências 7, 8 e 9 se referem aos estudos realizados, não devem ser generalizadas. Deve-se contextualizar os estudos, onde foram realizados com qual população e o seu resultado.

Página 4, último parágrafo, primeira frase? Citar a fonte da afirmativa.

Página 5. Primeiro parágrafo, a primeira frase necessita da citação. A segunda frase menciona estudos, porém somente uma referência foi citada.

Página 5, terceiro parágrafo está solto, precisa estabelecer as conexões.

Último parágrafo, a justificativa deve ser elaborada pelos(as) próprios(as) autores(as). Na justificativa deve-se informar a relevância do estudo, a sua contribuição e a inovação do estudo. As referências devem ser removidas da introdução.

A frase sobre as poucas evidências, permanece, embora os(as) autores(as) tenham afirmado que foi removida. Esse tipo de afirmativa, necessita de um estudo de revisão e esse não foi o caso.

Métodos

O nome do projeto pode ser removido e apresentado nos agradecimentos. De fato, o projeto não faz parte do desenho do estudo.

No contexto além de apresentar as comunidades é importante informar o motivo da sua escolha para o estudo.

As entrevistas devem ser apresentadas no item instrumentos de pesquisa. Solicitado anteriormente, embora os(as) autores(as) informem ter realizado permanece sem alteração.

Todas as variáveis devem ser acompanhadas das suas categorias.

O item instrumentos de pesquisa contêm a descrição de algumas variáveis. Sugere-se que todas as variáveis, juntamente com as suas categorias, sejam descritas no item variáveis.

Os métodos estatísticos precisam ser descritos de forma didática e compreensível. A seção está iniciando com o intervalo de confiança. Sugere-se que seja descrita na forma que foi realizada, buscando tornar a seção compreensível.

Resultados

Os resultados do primeiro parágrafo não foram localizados nas tabelas.

Resultados não devem ser interpretados (aproximadamente dois terços).

Observa-se que tem resultados que estão nas tabelas e não constam nos resultados. Os resultados da tabela 2 não foram citados no texto. A tabela 2 está sendo citada na discussão. As duas seções precisam ser revisadas com extrema atenção.

As limitações do estudo devem anteceder a conclusão.

Observa-se a presença de parágrafos que precisam estabelecer a conexão com os resultados. Estão parecendo revisão de literatura.

Discussão

Observa-se a presença de dados numéricos dos resultados na seção. Revisar para não incluir ou repetir resultados.

A discussão deve seguir a mesma sequência de apresentação dos resultados. Vários resultados foram apresentados nas tabelas, não estão descritos na seção de resultados, mas estão sendo discutidos. Importante revisar para que todos os resultados sejam descritos na seção correspondente.

Referências

A referência 2 não está citada corretamente.

Observa-se a presença de referências que necessitam de atualização.

Comentários menores

A revisão técnica indicada, deve ser preservada durante a realização das modificações sugeridas.

Nos intervalos de confiança, usar sempre o modelo “(IC95% X,X;X,X)” ou “(IC95% X,XX;X,XX)”, com espaço apenas após o “95%”.

Parágrafos 1 e 2 da página 4, iniciando da mesma forma.

A redação precisa ser revisada e os adjetivos devem ser removidos.

Indicar os cardinais por extenso de um a dez. Nos demais casos, usar algarismos. Revisar o segundo parágrafo da página seis.

O manuscrito necessita de revisão linguística.

Recommendation: Minor revision

## Seventh round comments

Estimadas autoras e estimados autores

O Núcleo Editorial da revista Epidemiologia e Serviços de Saúde (RESS), após a análise da reformulação do manuscrito intitulado “Fatores associados ao controle pressórico inadequado em hipertensos residentes em comunidade quilombola do Nordeste do Brasil, de 2017 a 2018: estudo transversal” (RESS-2024-0556.R6), sugere, ainda, que sejam realizadas adequações.

Todos os comentários e sugestões devem ser considerados e respondidos, item por item em carta aos editores(as). Para as sugestões que forem atendidas, deverá ser realçada a alteração realizada no manuscrito em amarelo. As sugestões que não forem consideradas deverão ser justificadas com fundamentação científica. A versão reformulada do manuscrito deve considerar as instruções aos autores da RESS e todos os itens de padronização indicados na revisão técnica. Solicita-se, gentilmente, que os(as) autores(as) não deixem para enviar o manuscrito no último dia, pois o sistema não é nacional e sempre irá expirar o prazo no dia limite.

Cordialmente,

Núcleo Editorial

Epidemiologia e Serviços de Saúde: revista do Sistema Único de Saúde do Brasil

Site: www.scielo.br/ress

Introdução

A introdução permanece extensa, foi ampliada em relação à versão anterior. Observa-se a manutenção de informações repetidas. Por favor, revisem a introdução buscando sintetizá-la.

Página 4, segundo parágrafo - incluir a referência que fundamenta a última frase.

Página 4, terceiro parágrafo - primeira frase especificar quais são os diversos obstáculos e incluir a referência.

Página 4 - frase que inicia na linha 27, incluir a referência que fundamenta a afirmativa.

Página 5, primeiro parágrafo, primeira linha incluir referência.

Página 5, segundo parágrafo - necessita incluir as referências que fundamentam as afirmativas.

Página 5 - linha 34 - parágrafo de frase única.

Método

No item contexto. Sugere-se a retirada do vocábulo “grande”.

No item “participantes” - Sugere-se a retirada do vocábulo “real”.

Página 7 - substituir dez por 10

Resultados

Primeiro parágrafo, citar a tabela na qual se encontram os resultados.

Para apresentação das frequências absolutas e relativas utilizar o seguinte formato: (n=215; 71,7%).

Discussão

Remover todos os dados numéricos de resultados e a citações das tabelas.

Os três últimos parágrafos da página 10, precisam estabelecer a conexão com os resultados do estudo. A forma atual traz apenas a literatura. Observa-se ainda que dois desses parágrafos são constituídos por uma única frase.

Conclusão

Remover a expressão “em resumo”.

Recommendation: Minor revision

